# Use of the Prompts for Reestructuring Oral Muscular Phonetic Targets (PROMPT) in Autism Spectrum Disorder: a case study

**DOI:** 10.1590/2317-1782/20232022299en

**Published:** 2023-12-18

**Authors:** Denise Miranda de Oliveira Donadio, Marcia Simões-Zenari, Thaís Helena Ferreira Santos, Maria Gabriela Sanchez, Daniela Regina Molini-Avejonas, Daniela Cardilli-Dias

**Affiliations:** 1 Programa de Pós-graduação em Ciências da Reabilitação, Faculdade de Medicina, Universidade de São Paulo – USP - São Paulo (SP), Brasil.; 2 Faculdade de Medicina, Universidade de São Paulo – USP - São Paulo (SP), Brasil.; 3 Laboratório de Investigação Fonoaudiológica de Saúde Mental, Faculdade de Medicina, Universidade de São Paulo – USP - São Paulo (SP), Brasil.; 4 Universidade de Ciencias Médicas, Universidad Nacional de Córdoba - Córdoba, Argentina.

**Keywords:** Autistic Disorder, Child Language, Articulation Disorders, Language Therapy, Rehabilitation of Speech and Language Disorders, Phonoaudiology

## Abstract

Autism Spectrum Disorder (ASD) is classified by Diagnostic and Statistical Manual of Mental Disorders (DSM-5) as a neurodevelopmental disorder, whose characteristics are mainly deficits in social communication and a restricted range of interests. There are several studies about autism, speech, and language in the literature, but few correlate speech and autism. This study aims to carry out a case study that will address autism, speech, and PROMPT (Restructuring Oral Muscular Phonetic Targets) and also to describe the speech improvement in the participant with autism using the method. The target words were defined for the entire intervention according to the System Analysis Observation (SAO) and Motor Speech Hierarchy (MSH), which are parts of the PROMPT evaluation. After the evaluation, the participant was attended for 16 sessions, once weekly, with the objective of improving their speech. After analyzing the data, it was possible to observe improvement in all aspects outlined according to the pre-treatment evaluation of the method such as phonatory control, mandibular control, lip-facial control and lingual control as well as in the sequenced movement although this was not the aim outlined in the evaluation. It was also possible to measure the improvement of an adequate number of words, an adequate number of phonemes, percentages of correct consonants – revised (PCC-R), and intelligibility.

## INTRODUCTION

PROMPT was developed by the speech therapist Déborah Hayden and colleagues in the late 1970s based on the absence or lack of response of patients with speech disorders, either of an acquired or developmental nature, to traditional treatment approaches predominantly based on strategies focused on hearing or visual routes.

PROMPT stands for Prompts for Restructuring Oral Muscular Phonetic Targets. It is considered a philosophy, an approach, a system, and a technique that includes neuromotor principles, auditory, visual, and somatosensory (kinesthetic and proprioceptive) information to provide feedback to the speech system.

The PROMPT therapy is guided by the “PROMPT Conceptual Framework”, which suggests that global domains, including physical, mental, and emotional, are interdependent and develop along a continuum of normal communication skills^(1)^.

The PROMPT Conceptual Framework and Motor-Speech Hierarchy (MSH) are described as structures for both validation and treatment that help speech therapists develop a holistic communication focus while incorporating motor, language, and social interaction goals. The PROMPT role of the technique and tactile systems have relevant use and application in children who have moderate to severe mixed phonological and motor impairment^(2)^.

The System Analysis Observation (SAO) and MSH are the two instruments used for the PROMPT assessment. The SAO is a measure designed to assess structure, function, and integration by showing the functioning of the patient’s motor subsystems when speech is produced. The total of answers “NO” in the SAO establishes the participant’s evolution. If the answer is “YES”, the item is as expected. If the answer is “NO”, there is a need for treatment^(1)^.

MSH is used to assess the motor speech system systematically and detect the levels or stages of difficulties. It identifies seven stages of speech development and motor control. These stages are considered hierarchically dependent and interactive, directly influencing the development of the next stages^(2)^.

The PROMPT approach is an intervention based on tactile-kinesthetic points to treat motor speech disorders by facilitating the place, manner, and time of speech movements by precise tactile-kinesthetic application to the patient’s face^(3)^.

Originally created to enhance and restructure oral muscle phonetic targets of speech, the PROMPT has evolved into a complete model of assessment and treatment^(4)^.

**Figure 1:**
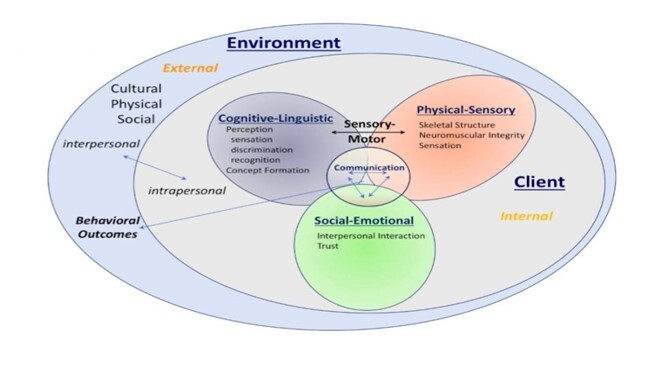
PROMPT Conceptual Framework^(1)^

According to the PROMPT Conceptual Framework, if any of these global domains is disorganized, delayed, or impaired, speech production will not develop regularly. The PROMPT system of assessment and treatment of individuals incorporates all these domains. PROMPT covers not only speech but all aspects of the patients must be considered, according to the model^(1)^:

As shown in Figure 1, the ability to accurately perceive spoken language is a fundamental skill in social communication. Speech perception, although often conceptualized as an auditory process, is inherently multisensory, with a listener using both auditory speech information and visual speech information in the form of oral articulations^(5)^.

If infants and children with Autistic Spectrum Disorder (ASD) are considerably less inclined to fixate properly on a speaker, they may simply receive much less cross-sensory learning experience throughout their early years. Thus, critical cross-sensory correspondences will not be encoded properly or deeply^(6)^.

More than a practical method of speech correction, the philosophy and conceptual structure of the PROMPT cover the whole communication action, including the physical-sensory, cognitive-linguistic, and emotional-social domains that develop and interact in normally developing humans^(4)^.

This case study aimed to measure how the PROMPT improves the speech of the child participant with ASD. This is the first case study describing children with ASD who are Brazilian Portuguese speakers using this method.

## CLINICAL CASE PRESENTATION

This study was approved by the Ethic Research Committee of the University of São Paulo – Decision Number 3.689.420.

The case describes a male patient with ASD aged six years old at the beginning of the study. Since the participant was diagnosed prior to the research, it was not possible to measure the support level as the information was not in the medical records. Prior to the start of the study, the patient’s caretakers were instructed about the research objectives and data collection and signed the Informed Consent Form.

We carried out an informal anamnesis gathering the participant’s information and an assessment of data collection regarding motor development, feeding, and speech development. The assessment included filming for word analysis, the Brazilian Standardized Test ABFW^(7)^, and the PROMPT assessment, which encompasses the SAO and MSH. We found no difficulty with the tactile touch on the participant’s face.

The ABFW language test was created for the Brazilian context and is composed of subtests that assess different areas involved in the communication process: phonology, vocabulary, fluency, and pragmatics. This case study assessed the patient only in the phonology subtest^(8)^.

The SAO revealed and measured the structural and skeletal aspects, in addition to assessing features of tone, valve, phonation, mandibular, labio-facial, and lingual controls, as well as sequenced movements and prosody.

MSH was originally developed to help conceptualize the various levels of the motor system that must be controlled to produce regular speech. The following levels were assessed: Level 1: tone, Level 2: phonatory control, Level 3: mandibular control, Level 4 labio-facial control, Level 5: lingual control, Level 6: sequenced movements, and Level 7: prosody^(9)^.

The assessment showed that the participant presented unintelligible connected speech during spontaneous speech productions, including a limited phonetic inventory of consonants, little lip contraction, and retraction, presenting immature mandibular control and mandibular lateral sliding.

The participant received 16 weekly PROMPT therapy sessions of 45 minutes each, in person. All sessions were held in the private office of the speech therapist responsible for this research. The initial and final sessions were recorded to enable greater detail in the analysis of the participant’s productions.

Following the collection of assessment data through the SAO and MSH, the target words were defined. The participant was given lexical planning appropriate to their assessment. In the first moment, many practices were carried out during the session and once the target word was produced, it was inserted in practices distributed in the reinforcing activities.

At the beginning of the treatment, we performed massive practice more intensively. Once the acquisition of target words was reached, we proceeded with distributed practices, and other words were selected when the participant could perform them correctly most of the time (around 80% success rate).

The judges received the recordings of the first session, which were then phonetically transcribed. Along with the recording, a collection video of the phonology naming test of the ABFW test plus a spontaneous speech span of the participant was also sent. The same procedure was performed after 16 sessions. The examiners tabulated the number of correct words, number of correct phonemes, and Percentage of Correct Consonants – Reviewed (PCC-R)^(10)^. The phonological analysis was carried out by two speech therapist examiners recommended by the research supervisor.

We used as analysis base the mean values from the analysis performed by the examiners, in addition to conducting the t-Student test to compare the performance before and after the intervention. The significance level adopted was 95%. The significant variables were marked as (*).

The analysis of the MSH protocol involved the analysis of each domain individually.

The acquisition of new words was analyzed in two steps. The first step is to set the targets for the participant according to the initial assessment. Once the goal was reached, new targets were set in the second step. The data describes both steps.

The literature points out the use of the Percentage of Correct Consonants (PCC) index to establish the severity of the phonological alteration qualitatively. The PCC considers omissions, substitutions, and distortions as errors and is recommended for children with phonological disorders between three and six years of age. However, to compare speakers of different ages and with different speech characteristics, the PCC-R was proposed, considering only substitutions and omissions as errors^(11)^.

According to Table 1, the participant presented higher numbers of words, phonemes, and PCC after the PROMPT intervention.

**Table 1:** Participant’s phonological performance analysis

**Phonological analysis**	**Before**	**After**
Number of correct words*	9	15
Number of correct phonemes*	8	18
PCC-R*	52%	78%

* variables showing significant evolution, p ≤  0.05 - t-Student.

**Caption:** PCC-R = Percentage of Correct Consonants – Revised.

**Figure 2:**
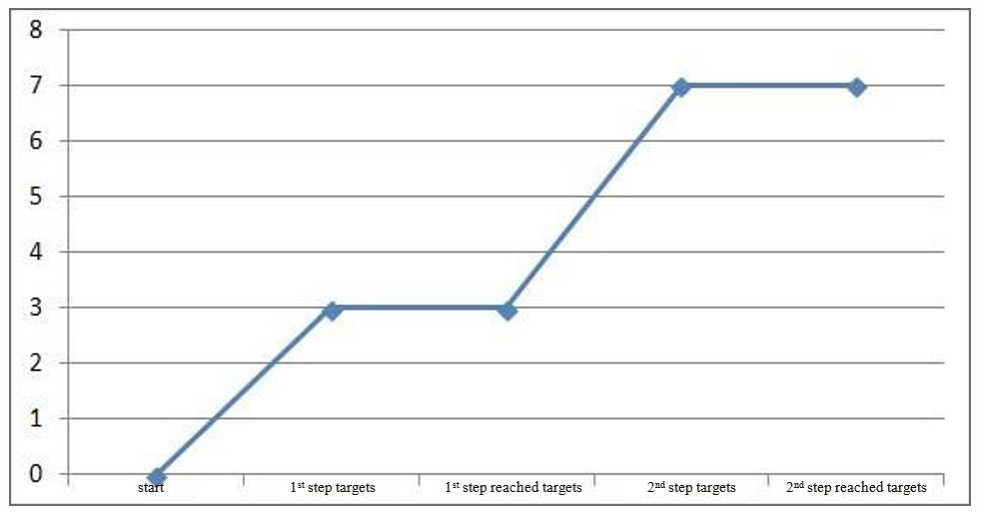
The participant’s performance chart for the set and reached targets

Figure 2 shows the targets that were set for the intervention with the participant according to the assessment – lasting the whole research period; however, the participant reached the goal before the end. Hence, new targets were established.

Table 2 shows the participant’s performance in the SAO assessment, which measured the moments before and after the treatment. Speech, phonatory control, mandibular control, labio-facial control, and lingual control, which were the targets, presented a lower number of answers “NO”, hence improving during the treatment. Even though the sequenced movement was not a target, it also showed improvement, pointing out that better levels of MSH can influence motor speech sequence.

**Table 2:** The participant’s performance analysis regarding the answer “no” for the System Analysis Observation (SAO)

**SAO analysis**	**Before**	**After**
Skeletal	2	2
Neuromotor function	1	1
Phonatory control	3	1
Mandibular control*	2	1
Labio-facial control*	5	3
Lingual control*	4	3
Sequenced movements*	5	4
Prosody	1	1

* categories showing significant evolution, p ≤  0.05 - t-Student.

Table 3 shows more detailed results such as the number of each speech subsystem, evolution in percentage, and number of target words that were planned and reached.

**Table 3:** The participant’s evolution in the analyzed areas.

**Participant’s analyses**		**Before**	**After**	**% Evolution**
**Motor hierarchy**	Skeletal	2	2	-
	Neuromotor function	1	1	-
	Phonatory control*	3	1	66%
	Mandibular control*	2	1	50%
	Labio-facial control*	5	3	40%
	Lingual control*	4	3	25%
	Sequenced movements*	5	4	20%
	Prosody	1	1	-
**Phonology**	Number of correct words*	9	15	83%
	Number of correct phonemes*	8	18	112.5%
	PCC-R*	52%	78%	26%
**Words -Target**	1^st^ step*	3 (targets)	3 (reached)	100%
	2^nd^ step*	4 (targets)	4 (reached)	100%
	Total*	7 (targets)	7 (reached)	100%

* categories showing significant evolution, p ≤  0.05 - t-Student; %: percentage.

**Caption:** PCC-R = Percentage of Correct Consonants – Revised.

## DISCUSSION

Despite being the first case study in Brazilian Portuguese analyzing a child with ASD using PROMPT, our findings proved to be significant and corroborated the efficacy demonstrated in prior international studies.

A 2020 randomized control study in children with severe motor speech delay reported that the PROMPT intervention is an effective approach for such a population, with the children presenting improved motor speech control, articulation, and speech intelligibility in the word^(12)^.

A 2021 study conducted on ten children with childhood apraxia of speech where five children received PROMPT treatment and five children received conventional treatment found significant improvements in the area of sequencing and connected speech, with better sentence production, significantly increased word accuracy, diadochokinesis for three-syllable sequences, and speech intelligibility. The group that received conventional treatment showed improvement in phonetic inventory and diadochokinesis for two-syllable sequences^(13)^.

Our results demonstrate that the number of correct words, adequate phonemes, and PCC-R increased significantly between the assessments before and after the intervention, thus improving word production.

The values from the MSH showed an evolution of 66% in phonatory control, 50% in mandibular control, 40% in labio-facial control, and 25% in lingual control. Thus, all domains set for the method showed some evolution.

## FINAL COMMENTS

As to the PROMPT assessments before and after the treatment, we found that the participant presented improved phonatory control, mandibular control, labio-facial control, and lingual control. In addition, despite not being our target, the sequenced movements also improved. We conclude that PROMPT is an effective treatment in improving the aspects of motor speech control in an autistic child.

We suggest a long-term follow-up to measure the generalization of the work and study involving more participants at different ages and with varied ASD support levels.

## References

[1] Hayden D. (2012). Manual de introdução à técnica PROMPT..

[2] Hayden D (2006). The PROMPT model: use and application for children with mixed phonological-motor impairment. Adv Speech Lang Pathol.

[3] Namasivayam AK, Pukonen M, Goshulak D, Yu VY, Kadis DS, Kroll R (2013). Relationship between speech motor control and speech intelligibility in children with speech sound disorders. J Commun Disord.

[4] Hayden D, Stockman IJ (2004). Movement and action in learning and development: clinical implications for pervasive developmental disorders..

[5] Stevenson RA, Baum SH, Segers M, Ferber S, Barense MD, Wallace MT (2017). Multisensory speech perception in autism spectrum disorder: from phoneme to whole-word perception. Autism Res.

[6] Foxe JJ, Molholm S, del Bene VA, Frey HP, Russo NN, Blanco D (2015). Severe multisensory speech integration deficits in high-functioning school-aged children with Autism Spectrum Disorder (ASD) and their resolution during early adolescence. Cereb Cortex.

[7] Wertzner HF, Andrade C, Befi-Lopes D, Fernandes F, Wertzner H (2004). ABFW Teste de linguagem infantil nas áreas de fonologia, vocabulário, fluência e pragmática..

[8] Carbonieri J, Lúcio PS (2020). Avaliação do vocabulário em crianças brasileiras: revisão sistemática de estudos com três instrumentos. CoDAS.

[9] Hayden DA, Square PA (1994). Motor speech treatment hierarchy: a systems approach. Clin Commun Disord.

[10] Campbell TF, Dollaghan C, Janosky JE, Adelson PD (2007). A performance curve for assessing change in Percentage of Consonants Correct Revised (PCC-R). J Speech Lang Hear Res.

[11] Befi-Lopes DM, Tanikawa CR, Cáceres AM (2012). Relação entre a porcentagem de consoantes corretas e a memória operacional fonológica na alteração específica de linguagem. Rev Soc Bras Fonoaudiol.

[12] Namasivayam AK, Huynh A, Granata F, Law V, van Lieshout P (2021). PROMPT intervention for children with severe speech motor delay: a randomized control trial. Pediatr Res.

[13] Fiori S, Pannek K, Podda I, Cipriani P, Lorenzoni V, Franchi B (2021). Neural changes induced by a speech motor treatment in childhood apraxia of speech: a case series. J Child Neurol.

