# ERRATUM: Use of the Prompts for Reestructuring Oral Muscular Phonetic Targets (PROMPT) in Autism Spectrum Disorder: a case study

**DOI:** 10.1590/2317-1782/20242023279en

**Published:** 2024-05-27

Due to author's honest mistake the article “Use of the Prompts for Reestructuring Oral Muscular Phonetic Targets (PROMPT) in Autism Spectrum Disorder: a case study” (DOI https://doi.org/10.1590/2317-1782/20232022299en) published in CoDAS 2024;36(2):e20220299, was published with an error in the authorship order on page 1.

**In the list of authors, where it read:**

Denise Miranda de Oliveira Donadio^1^https://orcid.org/0000-0002-7551-2778

Marcia Simões-Zenari^2^https://orcid.org/0000-0003-0505-6655

Thaís Helena Ferreira Santos^3^https://orcid.org/0000-0003-2903-4952

Maria Gabriela Sanchez^4^https://orcid.org/0000-0002-4742-181X

Daniela Regina Molini-Avejonas^2^https://orcid.org/0000-0002-9768-882X

Daniela Cardilli-Dias^1^https://orcid.org/0000-0002-7615-7974

**It should read:**

Denise Miranda de Oliveira Donadio^1^https://orcid.org/0000-0002-7551-2778

Marcia Simões-Zenari^2^https://orcid.org/0000-0003-0505-6655

Thaís Helena Ferreira Santos^3^https://orcid.org/0000-0003-2903-4952

Maria Gabriela Sanchez^4^https://orcid.org/0000-0002-4742-181X

Daniela Cardilli-Dias^1^https://orcid.org/0000-0002-7615-7974

Daniela Regina Molini-Avejonas^2^https://orcid.org/0000-0002-9768-882X

The authors apologize for the errors.

