# Supplementary material for: Use of the Prompts for Reestructuring Oral Muscular Phonetic Targets (PROMPT) in Autism Spectrum Disorder: a case study
Source: Codas. 2023 Dec 18;36(2):e20220299. doi: 10.1590/2317-1782/20232022299en (PMC10750890; doi:10.1590/2317-1782/20232022299en)
Supplement: Translation

# Translation: Uso do método de Pontos para a Reestruturação de Objetivos Fonéticos e Oro-Musculares (PROMPT) no Transtorno do Espectro Autista: um estudo de caso

**DOI:** 10.1590/2317-1782/20232022299pt

**Authors:** Denise Miranda de Oliveira Donadio, Marcia Simões-Zenari, Thaís Helena Ferreira Santos, Maria Gabriela Sanchez, Daniela Regina Molini-Avejonas, Daniela Cardilli-Dias

**Affiliations:** 1 Programa de Pós-graduação em Ciências da Reabilitação, Faculdade de Medicina, Universidade de São Paulo – USP - São Paulo (SP), Brasil.; 2 Faculdade de Medicina, Universidade de São Paulo – USP - São Paulo (SP), Brasil.; 3 Laboratório de Investigação Fonoaudiológica de Saúde Mental, Faculdade de Medicina, Universidade de São Paulo – USP - São Paulo (SP), Brasil.; 4 Universidade de Ciencias Médicas, Universidad Nacional de Córdoba - Córdoba, Argentina.

**Keywords:** Transtorno Autístico, Linguagem Infantil, Transtornos da Articulação, Terapia da Linguagem, Reabilitação dos Transtornos da Fala e da Linguagem, Fonoaudiologia

## Abstract

O Transtorno do Espectro Autista (TEA) é classificado pelo Manual de Diagnóstico e Estatístico de Transtornos Mentais (DSM-5) como um Transtorno do Neurodesenvolvimento, sendo caracterizado, principalmente, por alterações na comunicação social e pela presença de um repertório restrito de atividades e interesses. Na literatura, há muitos estudos sobre autismo, fala e linguagem, mas poucos correlacionando fala e autismo. Este estudo teve como finalidade realizar um estudo de caso que abordou autismo, fala e PROMPT (Pontos para a Reestruturação de Objetivos Fonéticos e Oro-Musculares) e mensurou a melhora da fala no participante com autismo, utilizando o método. Foram definidas palavras-alvo para toda a intervenção, conforme o Sistema de Observação e Análise (SOA) e Hierarquia Motora de Fala (HMF), que fazem parte da avaliação de PROMPT. Após a avaliação, o participante foi atendido por 16 sessões, uma vez semanalmente. Após análise dos dados, observou-se melhora em todos os aspectos que foram delineados de acordo com a avaliação no pré-tratamento, como controle fonatório, controle mandibular, controle lábio-facial e controle lingual, como também no movimento sequenciado, apesar deste não ser o objetivo traçado na avaliação. Também foi possível mensurar melhora no número de vocábulos adequados, número de fonemas adequados, porcentagens de consoantes corretas – revisado (PCC-R) e inteligibilidade.

## INTRODUÇÃO

O PROMPT foi desenvolvido pela fonoaudióloga Déborah Hayden e colegas no final da década de 70, motivados pela ausência ou pouca resposta dos pacientes, com transtornos de fala, de natureza adquirida ou desenvolvimental, às abordagens tradicionais de tratamento, baseadas predominantemente em estratégias focadas nas vias auditivas e visuais.

PROMPT é um acrônimo para Pontos para a Reestruturação de Objetivos Fonéticos e Oro-Musculares. É considerado uma filosofia, uma abordagem, um sistema e uma técnica. Esta inclui princípios neuromotores, informação auditiva, visual e somatoestésica (cinestésica e proprioceptiva) para fornecer uma retroalimentação (feedback) ao sistema de fala.

A terapia PROMPT é guiada pelo “Marco Conceitual de PROMPT”, o qual sugere que os domínios globais, incluindo físico, mental e emocional, são interdependentes e se desenvolvem ao longo de uma continuidade das habilidades normais da comunicação^(1)^.

O Marco Conceitual de PROMPT e a Hierarquia Motora da Fala (HMF) são descritos como estruturas tanto para avaliação quanto para tratamento que ajudam os fonoaudiólogos a desenvolver um foco holístico de comunicação ao mesmo tempo em que incorporam objetivos motores, linguísticos e de interação social. O papel da técnica e dos sistemas táteis no PROMPT é explicado com relevância para o seu uso e aplicação com crianças que têm comprometimento fonológico e motor misto de moderado a grave^(2)^.

Dois instrumentos são utilizados para a avaliação PROMPT, que são o Sistema de Observação e Análise (SOA) e a HMF. O SOA é uma medida projetada para avaliar a estrutura, bem como a função e a integração. Ele mostra como os subsistemas motores do paciente estão funcionando quando a fala é produzida. O total de “nãos” apresentado a partir do SOA foi considerado para determinar a evolução do participante. Se marcamos “SIM”, o item está dentro do esperado. Se marcarmos “NÃO”, indica-se que é uma área a qual necessita de tratamento e sugere-se que ela possa ser trabalhada^(1)^.

A HMF é utilizada para avaliar sistematicamente o sistema motor da fala e identificar os níveis ou estágios em que ocorrem as dificuldades. Ela identifica sete estágios de desenvolvimento de fala e controle motor. Esses estágios são considerados hierarquicamente dependentes e interativos e influenciam diretamente o desenvolvimento dos estágios sucessivos^(2)^.

A abordagem PROMPT é uma intervenção baseada em pontos táteis cinestésicos para trabalhar as desordens motoras de fala, em que o lugar, a maneira e o tempo dos movimentos da fala são facilitados pelo uso tátil-cinestésico preciso no rosto do paciente^(3)^.

Originalmente criado para aprimorar e reestruturar os alvos fonéticos musculares orais da fala, o PROMPT evoluiu para um modelo completo para avaliação e tratamento^(4)^.

De acordo com o Marco Conceitual de PROMPT, se qualquer um desses domínios globais está desorganizado, atrasado ou debilitado, a produção da fala não pode desenvolver-se normalmente. O sistema PROMPT de avaliação e tratamento de indivíduos incorpora todos esses domínios. O PROMPT não trata apenas a fala, ou seja, todos os aspectos do paciente devem ser considerados conforme modelo^(1)^:

Conforme foi possível observar na Figura 1, a capacidade de perceber com precisão a linguagem falada é uma habilidade fundamental na comunicação social. A percepção da fala, embora muitas vezes conceituada como um processo auditivo, é de fato inerentemente multissensorial, com um ouvinte usando tanto informações auditivas de fala quanto informações visuais de fala na forma de articulações orais^(5)^.

**Figura 1:** Marco Conceitual de PROMPT^(1)^

Se bebês e crianças com Transtorno do Espectro Autista (TEA) estiverem consideravelmente menos inclinados a se fixar adequadamente em um falante, eles podem simplesmente receber muito menos experiência de aprendizado intersensorial ao longo de seus primeiros anos e, por sua vez, as correspondências intersensoriais cruciais não serão codificadas de forma adequada ou profunda^(6)^.

Mais do que um método prático de correção da fala, a filosofia e a estrutura conceitual do PROMPT abordam todo o ato de comunicação, incluindo como os domínios físico-sensorial, cognitivo-linguístico e emocional-social se desenvolvem e interagem em humanos normalmente em desenvolvimento^(4)^.

O objetivo deste estudo de caso foi mensurar o benefício do PROMPT para melhora de fala da criança participante com TEA. Este foi o primeiro estudo de caso descrito em crianças com TEA falantes do português brasileiro utilizando este método.

## APRESENTAÇÃO DO CASO CLÍNICO

Este estudo obteve aprovação ética do Comitê de Ética em Pesquisa da Universidade de São Paulo - Número do Parecer: 3.689.420.

O caso descrito é de um participante do sexo masculino, com seis anos de idade no início do estudo e com TEA. Este diagnóstico foi dado ao participante antes da pesquisa, não sendo possível mensurar o nível de suporte, pois o mesmo não estava descrito no laudo médico. Os responsáveis foram esclarecidos a respeito dos objetivos da pesquisa e da forma de obtenção dos dados para somente então realizaram o aceite do Termo de Consentimento Livre e Esclarecido e início do estudo.

Foi realizada anamnese informal, com dados sobre o participante e avaliação na qual foram colhidos dados, como desenvolvimento motor, alimentação e desenvolvimento da fala. Na avaliação foram realizadas filmagens para análise de vocábulos, avaliação do Teste Padronizado Brasileiro ABFW^(7)^ e avaliação proposta pelo PROMPT a qual consta o SOA e a HMF. Durante a avaliação não foi observada nenhuma dificuldade com o toque tátil em sua face.

O teste de linguagem ABFW foi criado para o contexto brasileiro, sendo composto de subtestes que avaliam diferentes áreas envolvidas no processo de comunicação: fonologia, vocabulário, fluência e pragmática. Para este estudo de caso, o participante foi avaliado apenas no subteste de fonologia^(8)^.

No SOA, foi possível observar e mensurar aspectos estruturais e esqueletais e avaliar aspectos de tônus, controle de válvula e fonação, controle mandibular, controle lábio-facial, controle lingual, movimentos sequenciados e prosódia.

A HMF foi originalmente desenvolvida para ajudar a conceituar os vários níveis do sistema motor que devem ser controlados para produzir uma fala dentro da normalidade. Os sistemas avaliados foram: Nível I: tônus, Nível II: controle fonatório, Nível III: controle mandibular, Nível IV: controle labial-facial, Nível V: controle lingual, Nível VI: movimentos sequenciados e Nível VII: prosódia^(9)^.

Na avaliação foi possível observar que o participante apresentou fala encadeada ininteligível durante as produções de fala espontânea, com inventário fonético de consoantes limitado, pouca contração e retração labial, apresentando controle mandibular imaturo e deslizamento lateral mandibular.

O participante desta pesquisa recebeu 16 sessões semanais de terapia PROMPT com duração de 45 minutos cada, presencialmente. Todas as sessões ocorreram no consultório particular da fonoaudióloga pesquisadora deste estudo. As sessões iniciais e finais foram filmadas para possibilitar maior detalhamento na análise das produções do participante.

Após a coleta de dados da avaliação por meio do SOA e da HMF, foram definidas as palavras-alvo. O participante teve o planejamento lexical adequado à sua avaliação. No primeiro momento, foi realizado um grande número de práticas durante a sessão e após a palavra-alvo já ser produzida eventualmente, era inserida em práticas distribuídas nas atividades reforçadoras.

No início do tratamento, foi realizada prática massiva com mais intensidade e, posteriormente, com a aquisição das palavras-alvo, eram realizadas práticas distribuídas e outras palavras eram selecionadas quando o participante já era capaz de realizá-las corretamente na maioria das vezes (cerca de 80% de acerto).

Foram enviadas aos juízes as gravações da primeira sessão do participante e a partir deste material, foi realizada a transcrição fonética. Em conjunto com a gravação, foi enviado um vídeo de coleta da prova de nomeação da fonologia do teste ABFW e mais um tempo de fala espontânea deste participante. O mesmo procedimento foi realizado após as 16 sessões. Os juízes tabularam os valores de número de vocábulos adequados, número de fonemas adequados e Percentual de Consoantes Corretas - Revisado (PCC-R)^(10)^. A análise da fonologia foi realizada por dois juízes fonoaudiólogos, que foram sugeridos pela orientadora deste artigo.

Foram utilizados como base da análise os valores médios da análise realizada pelos juízes e realizado o teste t-Student para comparar o desempenho antes e depois da intervenção. O nível de significância adotado foi 95%. As variáveis significativas foram marcadas com (*).

Para a análise do protocolo da HMF, cada uma de suas áreas foi analisada individualmente.

A análise da aquisição de novos vocábulos foi dividida em duas etapas. Na primeira etapa, foram estabelecidos alvos para o participante de acordo com a avaliação inicial e, conforme alcançado o objetivo, novos alvos foram estabelecidos na segunda etapa. Os dados descrevem as duas etapas.

A literatura aponta o uso do índice de Porcentagem de Consoantes Corretas (PCC) para determinar a gravidade da alteração fonológica de forma qualitativa. O PCC considera como erro omissões, substituições e distorções e seu uso é indicado para crianças com distúrbio fonológico entre três e seis anos. Entretanto, para comparar falantes de diversas idades e com características de fala distintas, foi proposto o PCC-R, que considera como erro apenas as substituições e as omissões^(11)^.

De acordo com a Tabela 1, o participante apresentou maior número de vocábulos, maior número de fonemas e maior número de PPC, após a intervenção com PROMPT.

**Tabela 1:** Análise do desempenho do participante em fonologia

**Análises de Fonologia**	**Pré**	**Pós**
Número de vocábulos adequados*	9	15
Número de fonemas adequados*	8	18
PCC-R*	52%	78%

* variáveis que apresentaram evolução significativa, com p ≤  0,05 - t-Student.

**Legenda:** PCC-R = Porcentagem de Consoantes Corretas – Revisado.

Como relatado na Figura 2 foram traçados alvos para a intervenção do participante de acordo com a avaliação, que duraria todo o tempo da pesquisa, mas antes do término o participante atingiu a meta e então, foram colocados novos alvos.

**Figura 2:** Gráfico de desempenho do participante em relação aos alvos estabelecidos e alcançados

A Tabela 2 mostra como foi o desempenho do participante na avaliação do SOA, nela foi mensurado o pré e pós tratamento. A fonação, controle fonatório, controle mandibular, controle lábio-facial, controle lingual os quais eram os objetivos, apresentaram um menor número de respostas “não”, ou seja, obtiveram melhora durante o tratamento. O movimento sequenciado não era um objetivo traçado inicialmente, mas também apresentou melhora, mostrando que quando os níveis mais baixos da HMF apresentam melhora, podem influenciar na sequência motora da fala.

**Tabela 2:** Análise do desempenho do participante em relação às respostas “não” do Sistema de Observação e Análise (SOA)

**Análise do SOA**	**Pré**	**Pós**
Esqueletal	2	2
Função Neuromotora	1	1
Controle fonatório	3	1
Controle Mandibular*	2	1
Controle Lábio -Facial*	5	3
Controle Lingual*	4	3
Movimentos Sequenciados*	5	4
Prosódia	1	1

* categorias que apresentaram evolução significativa, p ≤  0,05 - t-Student.

A Tabela 3 mostra os resultados mais detalhados, com o número de cada subsistema de fala, a evolução em porcentagem e a quantidade de vocábulos-alvo que foram planejados e adquiridos.

**Tabela 3:** Evolução do participante nas áreas investigadas

**Análises do Participante**		**Pré**	**Pós**	**% Evolução**
**Hierarquia Motora**	Esqueletal	2	2	-
	Função Neuromotora	1	1	-
	Controle fonatório*	3	1	66%
	Controle Mandibular*	2	1	50%
	Controle Lábio -Facial*	5	3	40%
	Controle Lingual*	4	3	25%
	Movimentos Sequenciados*	5	4	20%
	Prosódia	1	1	-
**Fonologia**	Número de Vocábulos Adequados*	9	15	83%
	Número de Fonemas Adequados*	8	18	112,5%
	PCC-R*	52%	78%	26%
**Vocábulos -Alvo**	1ª etapa*	3 (alvos)	3 (alcançados)	100%
	2ª etapa*	4 (alvos)	4 (alcançados)	100%
	Total*	7 (alvos)	7 (alcançados)	100%

* categorias que apresentaram evolução significativa, p ≤  0,05 - t-Student; %: porcentagem.

**Legenda:** PCC-R = Porcentagem de Consoantes Corretas – Revisado.

## DISCUSSÃO

Embora este tenha sido o primeiro estudo de caso realizado no português brasileiro em uma criança com TEA utilizando PROMPT, os achados foram bastante significativos e corroboram com a eficácia mostrada em diversos estudos internacionais já realizados.

Em 2020 foi realizado um estudo de controle randomizado em crianças com atraso motor de fala grave, no qual foi mostrado que a intervenção de PROMPT é uma abordagem eficaz para essa população, onde as crianças apresentaram melhora no controle motor da fala, articulação e inteligibilidade de fala na palavra^(12)^.

Num estudo de 2021, realizado em dez crianças com apraxia de fala na infância onde 5 crianças receberam tratamento PROMPT e 5 crianças receberam o tratamento convencional, foram encontradas melhorias significativas na área de sequenciamento e fala conectada, com melhora em produções de frases, melhora significativa na acurácia da palavra, diadococinesia em trissílabas e inteligibilidade da fala. O grupo que recebeu tratamento convencional apresentou melhora no inventário fonético e na diadococinesia em dissílabas^(13)^.

Neste estudo foi possível observar a partir dos resultados obtidos, que o número de vocábulos adequados, fonemas adequados e PCC-R tiveram aumento significativo entre as avaliações pré e pós-tratamento, aumentando assim a melhora na produção das palavras.

Os valores obtidos na HMF mostraram uma evolução de 66% no controle fonatório, 50% em controle mandibular, 40% no controle lábio-facial e 25% no controle lingual, ou seja, todas as áreas que foram definidas para o trabalho com o método, apresentaram evolução.

## COMENTÁRIOS FINAIS

Em relação à avaliação PROMPT realizada pré e pós-tratamento, observou-se que o participante apresentou melhora em controle fonatório, controle mandibular, controle lábio-facial, controle lingual e, apesar de os movimentos sequenciados não serem o objetivo do trabalho, também apresentaram melhora. Concluiu-se que PROMPT foi eficaz como forma de tratamento para promover a melhora nos aspectos do controle motor da fala em uma criança autista.

Sugere-se um acompanhamento longitudinal para mensurar a generalização do trabalho realizado e estudo com mais participantes com diferentes idades e diferentes níveis de suporte do TEA.
