# Supplementary material for: ERRATUM: Use of the Prompts for Reestructuring Oral Muscular Phonetic Targets (PROMPT) in Autism Spectrum Disorder: a case study
Source: Codas. 2024 May 27;36(3):e20230279. doi: 10.1590/2317-1782/20242023279en (PMC11166031; doi:10.1590/2317-1782/20242023279en)
Supplement: Translation

# Translation: ERRATA: Uso do método de Pontos para a Reestruturação de Objetivos Fonéticos e Oro-Musculares (PROMPT) no Transtorno do Espectro Autista: um estudo de caso

**DOI:** 10.1590/2317-1782/20242023279pt

Devido ao erro honesto dos autores o artigo “Uso do método de Pontos para a Reestruturação de Objetivos Fonéticos e Oro-Musculares (PROMPT) no Transtorno do Espectro Autista: um estudo de caso” (DOI https://doi.org/10.1590/2317-1782/20232022299pt), publicado em CoDAS 2024;36(2):e20220299, foi publicado com um erro na ordem de autoria na página 1:

**Na lista de autores, onde se lia:**

Denise Miranda de Oliveira Donadio^1^https://orcid.org/0000-0002-7551-2778

Marcia Simões-Zenari^2^https://orcid.org/0000-0003-0505-6655

Thaís Helena Ferreira Santos^3^https://orcid.org/0000-0003-2903-4952

Maria Gabriela Sanchez^4^https://orcid.org/0000-0002-4742-181X

Daniela Regina Molini-Avejonas^2^https://orcid.org/0000-0002-9768-882X

Daniela Cardilli-Dias^1^https://orcid.org/0000-0002-7615-7974

**Leia-se:**

Denise Miranda de Oliveira Donadio^1^https://orcid.org/0000-0002-7551-2778

Marcia Simões-Zenari^2^https://orcid.org/0000-0003-0505-6655

Thaís Helena Ferreira Santos^3^https://orcid.org/0000-0003-2903-4952

Maria Gabriela Sanchez^4^https://orcid.org/0000-0002-4742-181X

Daniela Cardilli-Dias^1^https://orcid.org/0000-0002-7615-7974

Daniela Regina Molini-Avejonas^2^https://orcid.org/0000-0002-9768-882X

Os autores pedem desculpas pelos erros.
